# Estimation of effects of space radiation using frozen mouse ES cells in ISS

**DOI:** 10.1093/jrr/rrt217

**Published:** 2014-03

**Authors:** K. Yoshida, M. Hada, K. Eguchi-Kasai, T. Teramura, F. A. Cucinotta, T. Morita

**Affiliations:** 1 Osaka City University, Graduate School of Medicine, Osaka 545-8585, Japan; 2 National Institute of Radiation, Chiba 263-8555, Japan; 3 USRA, Division of Space Life Sciences, Houston, TX 77058, USA; 4 Kinki University, Faculty of Medicine, Osaka 589-8511, Japan; 5 NASA Johnson Space Center, Houston, TX 77058, USA

It is important to estimate the influence of space radiation on human body during a longer stay in space, including missions to International Space Station (ISS), the moon, or Mars. We use mouse embryonic stem (ES) cells, which have normal karyotypes to estimate the effects of space radiation. For this purpose, mouse ES cells have been launched to the Japanese experiment module called ‘KIBO’ in ISS and stored in a freezer (MELFI) at −95°C until the return to the Earth (return in five times in 3 years). After returning to the Earth, the DNA damages of space-radiated ES cells will be analyzed by detecting histone H2AX foci and their chromosomal aberrations. The ES cells can also be microinjected into normal embryos and cultured *in vitro*. The surviving embryos will be implanted into pseudo-pregnant mouse uteruses. Their development and the birth will be examined to estimate the effect of space radiation on mammalian development (Fig. 1).

**Fig 1.**
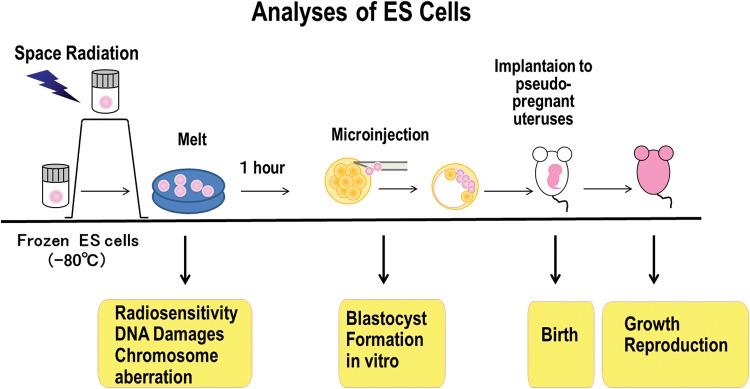
Outline of Space experiment. Mouse ES cells are frozen and kept in MELFI in ISS at −95°C from March 2013. Both wild-type and Histone H2AX-deficient ES cells are stocked. The frozen ES cells will return to the ground five times in 3 years. Their radiosensitivity, DNA damages and chromosome aberrations will be analyzed. The mouse ES cells will be then microinjected into normal embryos after 1 h culture. The blastocyst formation and hatching will be tested *in vitro*. Normally developed blastocysts will be implanted into pseudo-pregnant mouse uteruses and their birth and further growth will be examined to elucidate space radiation effects.

To analyze functions of DNA repair genes in space, we have also launched DNA repair-deficient mouse ES cells. Histone H2AX gene is involved in signaling and repair of DNA double-strand break, and the deletion of the gene will sensitize the ES cells to radiation. We have established mouse ES cells with heterozygous and homozygous deletion mutation of Histone H2AX by culture of the two-cell stage embryos derived from H2AX (+/−) heterozygous mouse mating. The heterozygous cells showed decreased H2AX protein level about half of that of wild-type (+/+). As control experiments on the ground, the ES cells were exposed to ^137^Cs γ-rays or 600 MeV/u ^56^Fe ions (LET of 173 keV/μm). Chromosome aberrations were analyzed by fluorescence *in situ* hybridization (FISH) technique with whole-chromosome probes during the first cell division after irradiation, and chromosome aberrations were identified as either simple exchanges (translocations and dicentrics) or complex exchanges (involving >2 breaks in two or more chromosomes).

Increased radiation sensitivity was observed in H2AX-heterozygous (+/−) and homozygous (−/−) cells compare with the wild type (+/+) toward both low- and high-LET radiation. Dose–response curves of chromosome exchanges in H2AX homozygous (−/−) and H2AX-heterozygous (+/−) cells were similar. H2AX homozygous (−/−) cells showed higher background level of exchanges, and higher frequency of break fragments compare with wild type (+/+) or heterozygous (+/−) cells, indicating its inability of rejoining the broken DNA. These H2AX gene deleted ES cells kept in ISS can be used for sensitive and quantitative estimation of biological influence of space radiation.

## FUNDING

This research was supported by partly “International Space Station (ISS) and Japanese Experiment Module “Kibo” Utilization Program” promoted by Japan Aerospace Exploration Abgency (JAXA).

